# uB-VisioGeoloc: An image sequences dataset of pedestrian navigation including geolocalised-inertial information and spatial sound rendering of the urban environment's obstacles

**DOI:** 10.1016/j.dib.2024.110088

**Published:** 2024-02-01

**Authors:** Florian Scalvini, Camille Bordeau, Maxime Ambard, Cyrille Migniot, Mathilde Vergnaud, Julien Dubois

**Affiliations:** a ImViA EA 7535 – Université de Bourgogne, Dijon, France; b LEAD CNRS UMR 5022, Université de Bourgogne, Dijon, France

**Keywords:** Pedestrian navigation, Virtual scene, Real scene, Camera RGB-D, GPS, IMU, Sonification, Artificial vision

## Abstract

The dataset proposed is a collection of pedestrian navigation data sequences combining visual and spatial information. The pedestrian navigation sequences are situations encountered by a pedestrian walking in an urban outdoor environment, such as moving on the sidewalk, navigating through a crowd, or crossing a street when the pedestrian light traffic is green. The acquired data are timestamped provided RGB-D images and are associated with GPS, and inertial data (acceleration, rotation). These recordings were acquired by separate processes, avoiding delays during their capture to guarantee a synchronisation between the moment of acquisition by the sensor and the moment of recording on the system. The acquisition was made in the city of Dijon, France, including narrow streets, wide avenues, and parks.

Annotations of the RGB-D are also provided by bounding boxes indicating the position of relevant static or dynamic objects present in a pedestrian area, such as a tree, bench, or person. This pedestrian navigation dataset aims to support the development of smart mobile systems to assist visually impaired people in their daily movements in an outdoor environment. In this context, the visual data and localisation sequences we provide can be used to elaborate the appropriate visual processing methods to extract relevant information about the obstacles and their current positions on the path. Alongside the dataset, a visual-to-auditory substitution method has been employed to convert each image sequence into corresponding stereophonic sound files, allowing for comparison and evaluation. Synthetic sequences associated with the same information set are also provided based on the recordings of a displacement within the 3D model of a real place in Dijon.

Specifications Table

**Table:** 

Subject	Computer Vision : Computer Science Applications
Specific subject area	Pedestrian viewpoint image sequences (real & synthetic) dataset with semantic and spatial metadata for computer vision research.
Data format	Raw colour and depth map images are in uncompressed format (.png). The camera motion files are given in a text file. The filtered annotation files are given in an XML file. The sonified videos are given in mkv format encapsulating a video stream encoded with a lossless x264 rgb codec and an audio stream encoded with a lossless FLAC codec.
Type of data	RGB-D image sequences from real and synthetic environments. Annotation of objects on the images. Recording of inertial and GPS sensors. Example of use of these data with sound samples.
Data collection	The dataset consists of two types of data: synthetic and real. The real data was collected using an onboard system comprising an IMU, GPS, and RGB-D sensors. A semi-qualified person carried out the annotation. The annotation was done using a semi-automatic approach combining deep learning techniques and human correction in a post-processing stage. On the other hand, the synthetic data was obtained by navigating in a virtual urban environment generated by a game engine.
Data source location	*· Institution : Université de Bourgogne* *· City/Town/Region : Dijon, Bourgogne-Franche Comté* *· Country: France* *· Latitude and longitude (and GPS coordinates, if possible) for collected samples/data: (47° 19′ 19.369" N 5° 2′ 29.328" E)*
Data accessibility	Repository name: Harvard Dataverse DOI: doi:10.7910/DVN/UYFPKM Direct URL to data: https://dataverse.harvard.edu/dataset.xhtml?persistentId=doi:10.7910/DVN/UYFPKM

## 1 Value of the Data

• The dataset is a collection of time-stamped synthetic and real annotated RGB-D image sequences from a pedestrian perspective.
• The real image capture was performed in the urban environment and associated with GPS and inertial data.
• The data contains examples of a visual-to-auditory encoding scheme for 3D visual scenes.
• The provided data can be used to apply the vision processing method to locate specific objects in an urban pedestrian area with a first-person point of view.
• The inclusion of inertial and GPS data enhances the video processing by providing information on the camera's geographic position, movement, and orientation.
• The research team could validate and compare a visual substitution device on the same data using the proposed dataset.

## 2 Data Description

**Fig. 1:**
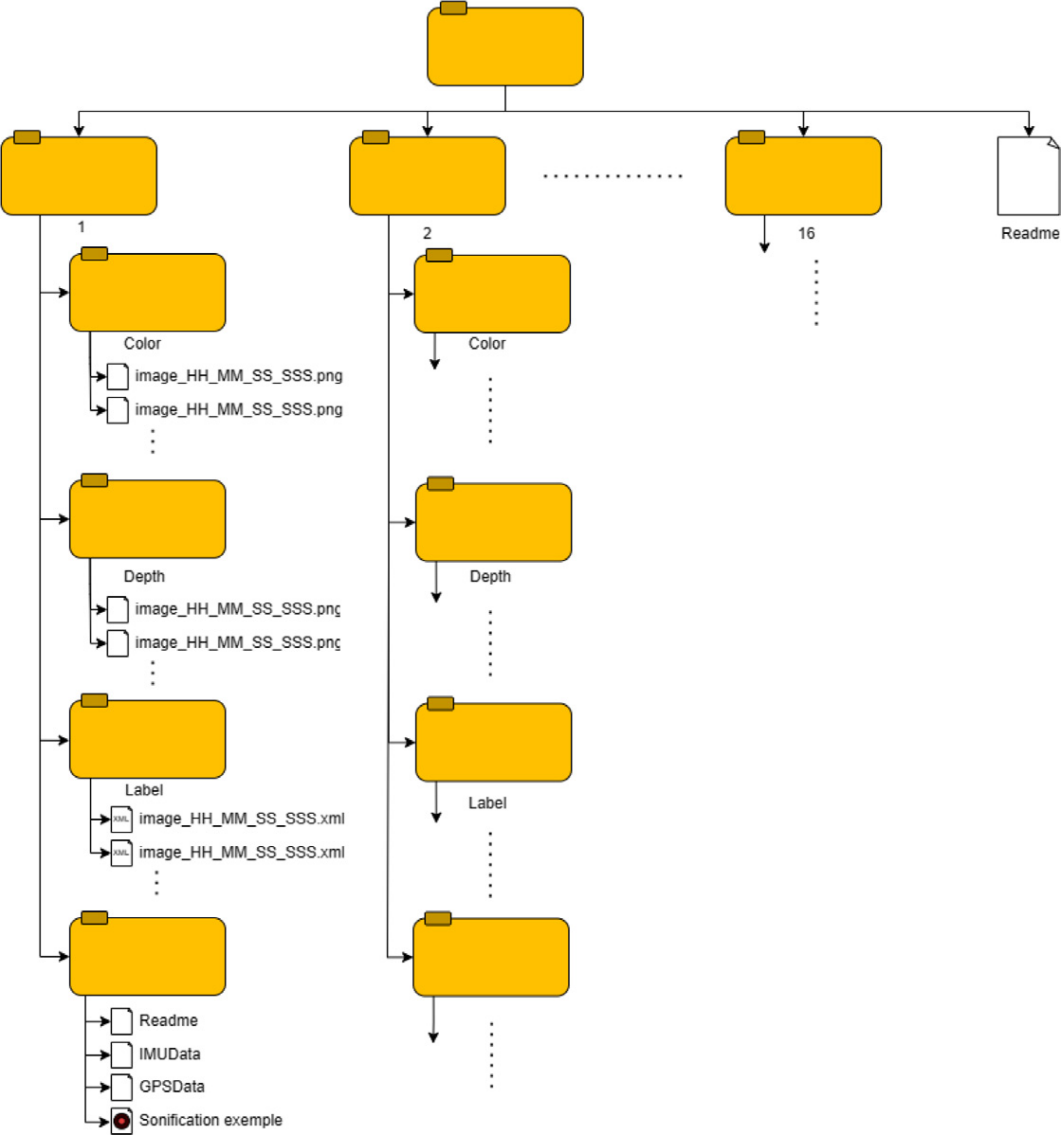
Dataset's organization.

**Fig. 2:**
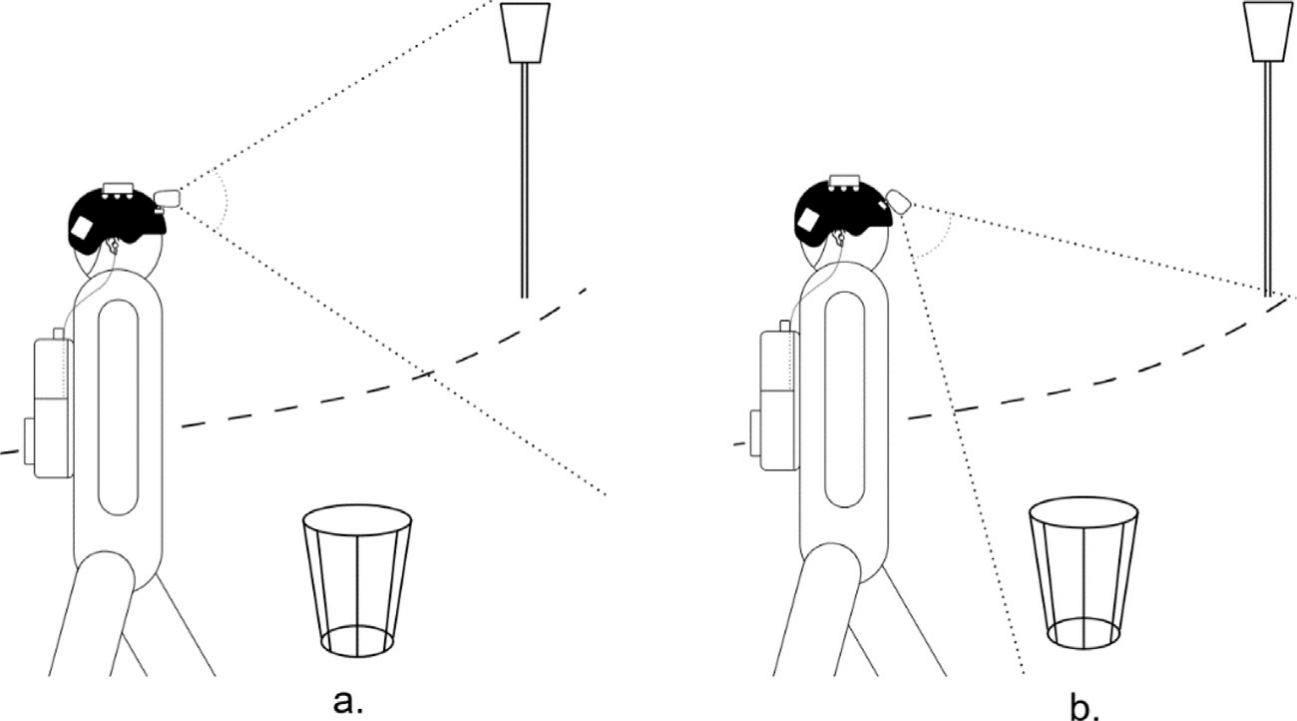
Schematic view illustrating various camera elevation positions. Fig. a corresponds to a 0° angle of elevation, while Figure b represents a -40° angle of elevation.

The purpose of this dataset is to provide a comprehensive collection of pedestrian navigation data sequences that reflect everyday life situations encountered in an urban environment. While most existing datasets primarily focus on the driver's perspective [1,2], this dataset aims to capture the pedestrian's point of view and their understanding of the surrounding environment during navigation. The dataset comprises 16 folders, one for each scene and one readme file (Fig. 1), with visual and spatial information. These scenes are collected separately in an urban environment, representing common situations encountered by individuals in everyday life when moving around outdoors and representing activities like crossing a road at a pedestrian crossing, moving through crowds, or walking along a pavement next to a road. While these situations may seem harmless, they pose significant challenges for autonomous robots or visually impaired individuals due to the diverse environments and varying levels of complexity. These scenes were acquired from real-world urban environments or generated using virtual simulations. Real and virtual environments play complementary roles in providing a comprehensive representation of the challenges faced during pedestrian navigation. The synthetic data, generated using a game engine, allows for precisely controlled and standardised representations of various obstacles. On the other hand, real-world data collected in actual urban environments offers a more diverse and authentic representation of challenges. Additionally, the dataset includes images captured from different camera elevation angles. Fig. 2 shows the varying field of view of the camera and its impact on scene acquisition. Specifically, a lower angular elevation prioritises nearby elements while limiting the maximum capture range. Table 1 gives a concise description of each image sequence, along with the nature of the data and the position of the elevation camera.

**Table 1:** Description of each image sequence identified by index number. The nature column indicates whether the scene is real or virtual. The elevation column provides details of the camera's angular position, with 0° representing a camera parallel to the ground, and the scene description column summarises each scene.

Scene	Nature	Elevation	Scene description
1	Synthetic	0°	A person crosses a road to enter a vast pedestrian place with various static obstacles. People are moving around the person while he continues walking on the place.
2	Synthetic	0°	A person is on a vast pedestrian place occupied by various static obstacles and moving persons. He walks 80m within this context while avoiding obstacles.
3	Real	-40°	A person strolling along the designated pedestrian path adjacent to a street, where cars and bicycles are parked.
4	Real	-40°	A pedestrian walking along the street on a path separated by a line
5	Real	-40°	A person walking on the sidewalk adjacent to a small street
6	Real	-40°	A person walking on the sidewalk adjacent to a small street
7	Real	-40°	A person walking on the sidewalk adjacent to a small street
8	Real	-40°	The pedestrian crosses a main road on a pedestrian crossing with pedestrian light traffic.
9	Real	-40°	A bus stops and picks up passengers before leaving
10	Real	-40°	A pedestrian area with tram tracks and a cobblestone path.
11	Real	-40°	A pedestrian crossing followed by a large footpath separated from the main road with many bikes and people.
12	Real	-40°	The pedestrian walks near a small street to join a place near a high school.
13	Real	0°	The pedestrian crosses an important road by multiple crossways.
14	Real	0°	The pedestrian walking along a path with bollards, cars, and people around him.
15	Real	0°	The pedestrian is walking in the city center with temporary signs and pedestrian crossing.
16	Real	0°	The pedestrian walks alongside a small street to join a place with multiple tables and chairs.

The image sequences, both real and simulated, provide a combination of visual, semantic, and time-stamped positional information from the pedestrian's perspective. The visual aspect is conveyed by RGB-D images that provide both colour and spatial information, detailed in Table 2. Semantic information indicates the location and nature of significant features in the images, while positional data provides information about the viewpoint and displacement of the recording environment. Additionally, audio files are associated with each data subset. These audio files represent the application of a sonification method, explicitly designed to aid visually impaired individuals in navigating unfamiliar environments. The audio provides an alternative sensory representation, allowing users to interpret and navigate the environment through sound.

**Table 2:** Description of data item.

Colour Image	A PNG image of the scene, with a 24-bit colour depth (3 bytes per pixel).
Depth Image	A PNG format depth image corresponding to the colour image's perspective, encoded in 16-bit grayscale (2 bytes per pixel).
Labels	<annotation> <filename>Color_HH_MM_SS_SSS.jpg</filename> <object> <name>3</name> <bndbox> <xmin>XX</xmin> <ymin>XX</ymin> <xmax>XX</xmax> <ymax>XX</ymax> </bndbox> </object> <object> <name>X</name> <bndbox> <xmin>XX</xmin> <ymin>XX</ymin> <xmax>XX</xmax> <ymax>XX</ymax> </bndbox> </object> </annotation>
IMU Data	HH;MM;SS;MMM; => Timestamp (Hours; Minutes; Seconds; Milliseconds) Compass; => Compass reading (value range from -180° to +180°) Qw;Qx;Qy;Qz; => Quaternion representing the user's head orientation Mx;My;Mz; => Magnetic field vector as measured by the magnetometer aLx;aLy;aLz => Linear acceleration vector (excluding gravity) Gx;Gy;Gz; => Gravity vector along x, y, and z axes
GPS Data	HH;MM;SS;MMM;Longitude;Latitude;
Sound file	A sonification example

**Fig. 3:**
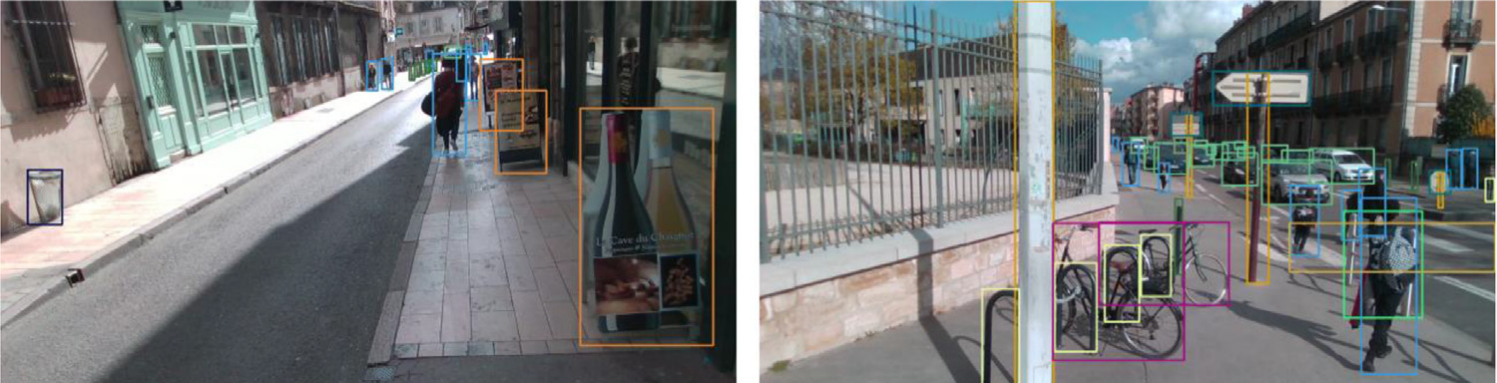
Illustration of image annotations.

The dataset consists of RGB images and depth maps recorded at a resolution of 1280 × 720 pixels and a frame rate of 30 frames per second. The limitations of the Intel RealSense RGB-D D435i camera used determined these specifications. The depth maps are stored as 16-bit images, representing a distance range of 0 to 65.535 meters. In these depth maps, one difference in light intensity corresponds to 1 millimetre of distance. This information provides a detailed spatial understanding of the scene, enabling accurate depth. Each RGB-D image is accompanied by an annotation file that contains semantic information about relevant elements present in the scene. These elements, categorised into 28 classes, include static and dynamic everyday objects in the pedestrian urban environment. The annotation file specifies the locations of these elements within the image by an axis-aligned 2D bounding box (Fig. 3).

*Dynamic Objects*: Car, Bus, Truck, Motorcycle, Scooter, Bicycle, Person, Animal.

*Static* objects: Bench, Dining Table, Chair, Fire Hydrant, Garbage, Traffic sign, Traffic light, Pole, Movable sign, Bus station, Fire hydrant, Pedestrian traffic light, Tree trunk, Crossway, Barricade, Bollard, Bike support, Potted plant, Parking meter, Border.

**Fig. 4:**
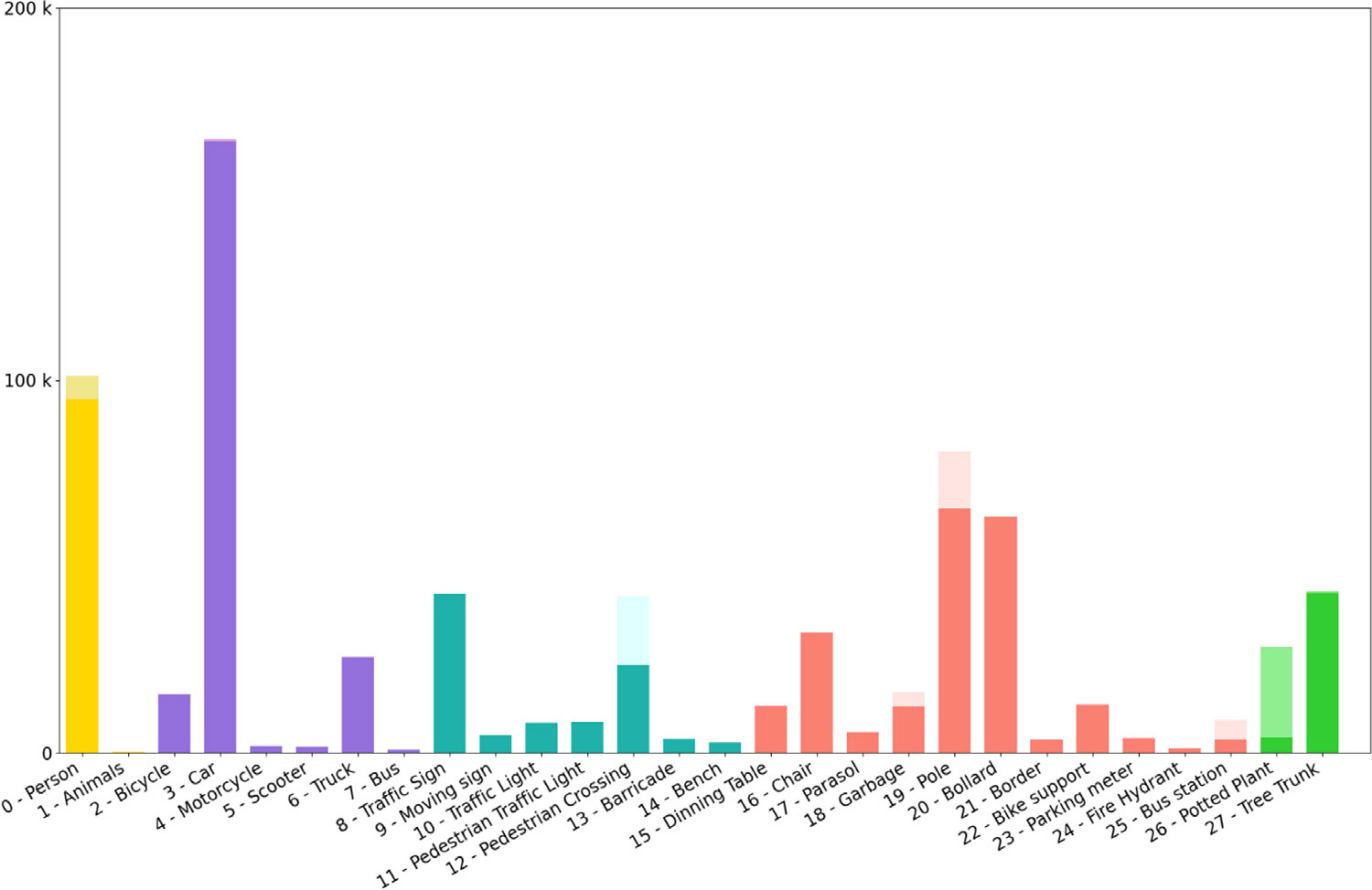
Class distribution in the dataset.

The relevant elements correspond to static or dynamic most common objects present in the pedestrian environment. The position of an object is represented by an axis-aligned 2D bounding box, obtained through a combination of automatic annotation and manual verification. The annotation file format follows the widely accepted Pascal VOC format used in object recognition datasets. Table 3 summarises the characteristics of the 16 image sequences within the dataset. Fig. 4 illustrates the occurrence of each class in the dataset, with colour indicating groups of classes sharing similar characteristics and colour nuances representing synthetic (Pastel colour, top of the column) or real data segments (bright colours, lower part).

**Table 3:** Distribution of the number of images and annotations in each set of recording images.

ID	Duration	Frames	Num. of annotation
1	55’	1636	43587
2	54’	1609	30996
3	30’	900	3759
4	30’	900	870
5	30’	900	2558
6	30’	900	8554
7	30’	900	13563
8	53’	1597	13472
9	30’	900	8511
10	26’	804	5449
11	30’	900	36677
12	184’	5522	173539
13	228’	6547	78591
14	285’	8579	40167
15	198’	5899	91848
16	311’	9344	184150

The dataset comprises an array of data captured from a human-centric viewpoint, detailing recorded positional coordinates, motion dynamics, and spatial orientation. This information is captured either directly in the virtual environment or by an IMU (Inertial Measurement Unit) sensor and GPS antenna in the recording system. The recorded data is stored in text files, with each line representing a set of time-stamped data separated by semicolons. Each line includes the recording time (in the format HH:MM:SS:SSS), the orientation of the viewpoint expressed as a quaternion, and the position information. The position is given in GPS coordinates. In contrast, for simulated scenarios, these GPS metrics are derived by calculating the tracked movements within the scene using an equirectangular projection technique to obtain the corresponding GPS coordinates. We also supply data concerning gravitational forces (measured in meters per second squared) and magnetic field strength (measured in microteslas).

## 3 Experimental Design, Materials and Methods

Despite the similar data formats, the data acquisition and annotation methods differed depending on whether the data came from virtual space or the real world. The synthetic data was generated by tracking the head of the participant wearing a virtual reality headset (Oculus Quest 2.) and simulating the corresponding video stream using the Unity Game Engine, and objects in the scene were automatically annotated based on their exact footprint on the rendered image. The real data was captured using an RGB-D camera and annotated using a convolutional neural network based on deep learning. This section explains the techniques used to acquire and annotate the videos and describes the method used to produce the accompanying sound files.

### 2.1 Real data

**Fig. 5:**
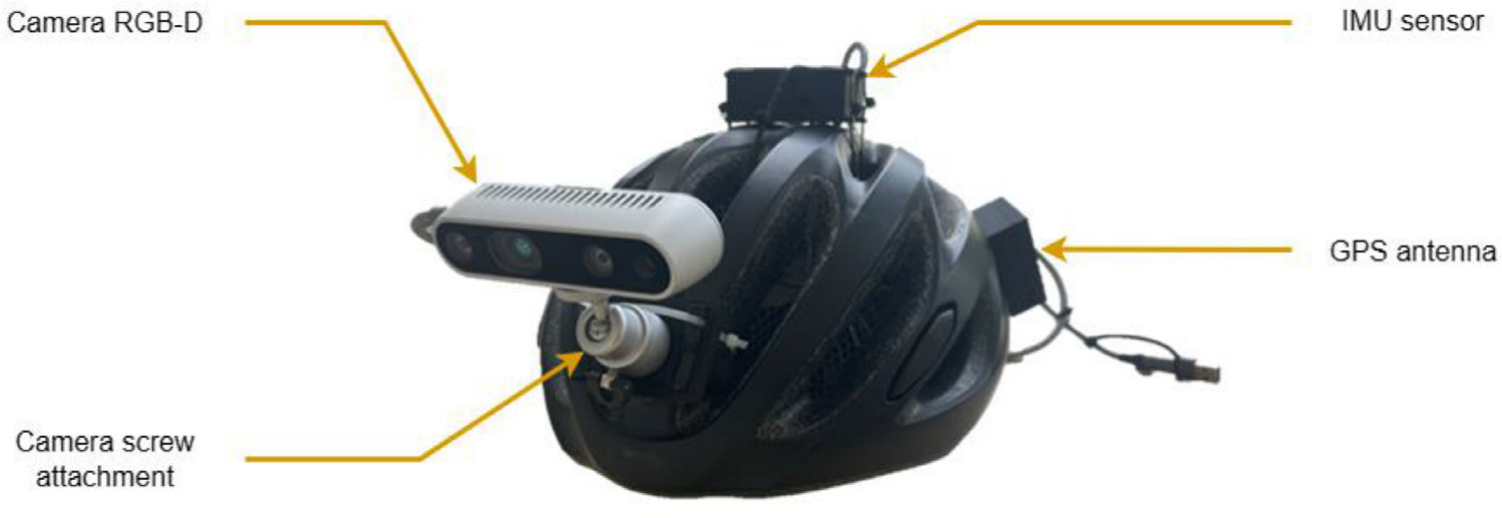
Experimental acquisition setup.

The data collection process in the city of Dijon involved a person equipped with a tracking system. The system, mounted on the user's helmet at a height of 1.85 meters, replicated the person's visual perspective. The camera's elevation was adjusted using the fixation screw, allowing the camera angle to be fine-tuned. The capture system, as shown in Fig. 5, consisted of several components. The primary component was the Intel Realsense D435 RGB-D camera, which captured colour (RGB) and depth (D) information at 30 frames per second. This camera was responsible for capturing the visual data as the person navigated. In addition to the camera, the acquisition system included an Adafruit BNO055 Inertial Measurement Unit (IMU) sensor and a GPS antenna, which recorded information about the environment at a frequency of 100 Hz and 10 Hz respectively. An Adafruit MCP2221A UART to USB module is also part of the device to facilitate communication between the IMU, GPS sensors, and the laptop. Data acquisition was performed by a C++ program using the Realsense 2 and OpenCV libraries. A multi-threaded approach ensured synchronisation between the data acquisition rate and the recording process. Each sensor had its dedicated thread running independently of the others. This design allowed each sensor to operate autonomously, unaffected by the performance of the other threads.

The annotation of the relevant elements of the urban scene according to the list of classes was generated using a semi-automatic method. This method involved a combination of pre-annotation by a convolutional neural network (CNN) and human annotation to ensure accurate labelling, including missing classes and correcting mislabelled elements. The pre-annotation stage utilised a YOLOv7 [3] architecture, which was trained on two distinct datasets. These datasets were carefully selected to cover everyday objects in urban areas. The MS COCO dataset [4] provided annotations for 80 common objects, encompassing outdoor items, animals, and more. On the other hand, the SideGuidedataset [5] specifically focused on pedestrian obstacles in a South Korean city. Additionally, a subset of annotated images from our dataset was used for training. However, a human correction and annotation step was applied to ensure the labels' accuracy. This manual process involved checking and adjusting the annotations frame by frame using a standard annotation tool.

### 3.2 Synthetic data

The synthetic environment models the popular Darcy place in Dijon, France. This low-poly virtual model was based on dot clouds acquired using a LiDAR scan (Light Detection And Ranging). The trajectory of the head was recorded by wearing a virtual reality headset (Oculus Quest 2.0) in an empty gymnasium. The 3D model and the head trajectories were reused in the 3DSmax software for optimal graphical rendering. The axis determines the bounding boxes of the objects that align a portion of the screen where the object is visible. To avoid the multiplicity of tiny bounding boxes of objects situated far from the camera but still visible on the screen, we only kept the objects less than 50m away and with a bounding box of more than 50 pixels. The same list of classes as for the real data was used to annotate the relevant elements. The annotation was generated automatically by a C# script running in Unity software using 3D virtual object labelling.

### 3.3 Sound generation

A visual-auditory encoding scheme was applied to the data set and associated audio files. The encoding scheme based on the monotone encoding of [6] consists of an image-processing step followed by image-to-audio conversion. Video processing extracts the intensity variation of pixel brightness by differentiating two successive depth maps previously converted to 8-bit images and resized between 0.2 m and 5.2 m with a resolution of 160 × 120 pixels. This range was chosen to focus on nearby elements that could endanger the visually impaired. The resulting image-sound conversion is based on the association of pixel position and brightness with a unique 3D spatialised sound, where the encoding scheme combines information on elevation, azimuth, and distance. The spatialised sound is a pure sound whose frequency depends on elevation (from 250 Hz to 1492 Hz), which is convolved with HRTFs from the CIPIC database [7] to obtain a stereophonic sound spatialised in azimuth and elevation. Finally, distance coding is added by modulating the intensity of the sound and envelope amplitude according to pixel brightness intensity. All the sounds generated are combined to produce an audio frame. The process is then repeated until the end of the image series to obtain a sequence of audio frames that generate an audio stream.

## Limitations

This data set has certain limitations that need to be considered. Data acquisition during pedestrian navigation required an onboard system where the sensors are connected via a USB connection to a laptop and introduced a potential for lost images (<5%). The presence of missing images is due to the preference for acquiring high-resolution images, which are more advantageous for the research team. These missing images, although corrupted, are included to avoid confusion. Moreover, the IMU accelerometer data included in the dataset exhibits drift over time, making it unsuitable in its raw form for accurately tracking the user's position over an extended period but may nevertheless allow displacement to be estimated over a short period. The specifications of the GPS device allow an accuracy of approximately 3 meters in clear weather. In densely populated urban areas, the accuracy of GPS is hindered by signal blockages caused by buildings, which challenges the precise pinpointing of a pedestrian's location. However, our navigation scenes take place mainly in open environments where the position of users is sufficiently approximate. This position can be refined, if necessary, by incorporating additional data sources such as visual cues. Some incorrectly labelled images can persist even if the data has been carefully annotated by several people who have examined the same image several times. On the other hand, the synthetic data from the dataset provides very accurate IMU and GPS information, enabling precise mapping on a 2D map of Dijon.

## Ethics Statement

Ethical considerations are paramount when dealing with a visual information data set acquired in a real urban environment. Indeed, the collection, dissemination and use of such data must comply with strict ethical rules to protect privacy. Our dataset is meticulously processed to ensure all personal identifiers, such as faces and vehicle license plates, are obscured in compliance with the ethical standards mandated for public space imagery in France. This involves blurring techniques to anonymise any potential identifiers captured during data acquisition. The French legislation stipulates the non-recognition of individuals in the foreground of any scene without their explicit consent. Therefore, when a person is relatively close to the camera, we slowly shift the camera's field of view to another element or the ground. In this way, the person's face was not recorded without their consent. In addition, the anonymisation process is carried out through an automated vision processing technique, ensuring the integrity and confidentiality of the subjects within the dataset.

## CRediT authorship contribution statement

**Florian Scalvini:** Conceptualization, Methodology, Software, Writing – original draft. **Camille Bordeau:** Conceptualization, Methodology, Writing – original draft. **Maxime Ambard:** Methodology, Software, Validation, Writing – review & editing. **Cyrille Migniot:** Writing – review & editing. **Mathilde Vergnaud:** Resources, Data curation. **Julien Dubois:** Writing – review & editing, Supervision.

## Data Availability

uB-VisioGeoloc: An image sequences dataset of pedestrian navigation including geolocalised-inertial information and spatial sound rendering of the urban environment’s obstacles (Original data) (Dataverse).
